# Double Pouch Colon Revisited

**Published:** 2014-01-01

**Authors:** Praveen Mathur, Mufique Gajdhar, Reyaz Ahmed, Arun K Gupta

**Affiliations:** Department of Pediatric Surgery, SMS Medical College, Jaipur, India

**Dear Sir**

A 3-day-old term male child weighing 2.1 Kg, born by spontaneous vaginal delivery, was admitted with complaints of abdominal distention, meconuria and absent anal opening. This was the first child of the couple who had a non-consanguineous marriage. On examination, there was abdominal distention, poorly developed gluteal folds and an absent anal opening. Plain X-ray abdomen showed two large air fluid levels (Fig. 1). At exploratory laparotomy, Saxena-Mathur classification type 5 congenital pouch colon (CPC) was found. Ileum was opening directly into a hugely distended pouch; there was no appendix. The pouch was dumbbell shaped with normal looking intervening colon of about 5 centimeters. The dumbbell shaped pouch (proximal and distal components) was opening into the bladder through a wide fistula. The proximal part of intervening colon was not communicating with the proximal pouch but was intimately adherent to it. However, the distal part of the intervening colon was communicating with the distal pouch. Both the dumbbell shaped pouch and the terminal colon were receiving blood supply from a prominent marginal vascular pattern (Fig. 2). The distal pouch and intervening normal-looking colon appeared dusky with doubtful viability, so it was excised. Tubularized proximal pouch was brought out as end colostomy. The histopathological examination revealed the disorganized muscles in the muscularis layer of pouch colon; the intervening colon had normal colonic histology though there was evidence of congestion. The child is awaiting definitive repair.

**Figure:**
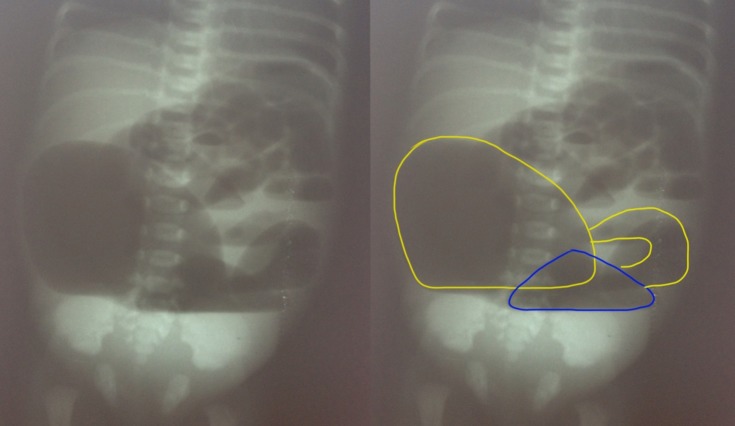
Figure 1: Radiological film demonstrating type 5 CPC.(Left- unedited; right- markings showing double pouch colon with short normal inter-positioned colon segment).

**Figure:**
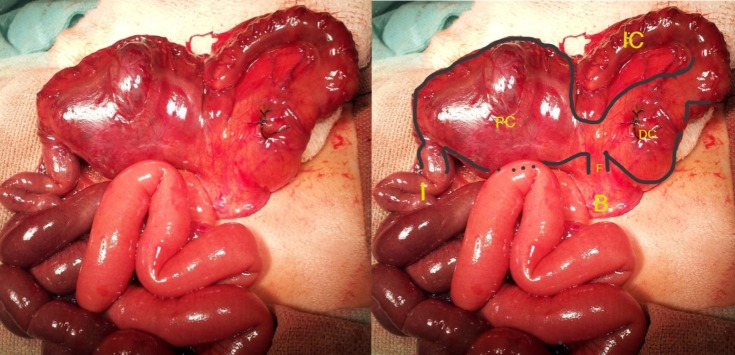
Figure 2: Operative photograph shows proximal and distal colonic pouches with intervening colon.(Left- unedited; right- edited with markings).

CPC is a rare variant of anorectal malformation (ARM) in which colon is foreshortened, with all or varying lengths of colon being replaced by a dilated pouch that is accompanied by a fistula, which communicates it with the genitourinary tract. The Krickenbeck classification for ARM has included it in the rare / regional variants group [1]. Most of the CPC large series have been reported from North Indian tertiary care centers. Udaipur in the Western State of Rajasthan has reported the highest incidence of CPC in India accounting for 37% of the high forms of anorectal malformations [2]. 

Based on the anatomic morphology of the pouch, the Saxena-Mathur classification differentiates five types of CPC [3].

Type 1 Normal colon is absent, and ileum opens into pouch colon.
Type 2 Ileum opens into a normal cecum that opens into pouch colon.
Type 3 Normal ascending colon and transverse colon open into pouch colon.
Type 4 Normal colon with rectosigmoid pouch
Type 5 Double pouch colon with short normal inter-positioned colon segment

The embryological and anatomical bases of formation of type 5 CPC have been previously described (4). This type of pouch is formed by defective embryogenesis on the basis of vascular insult theory proposed for CPC. According to them obliteration of ileocolic branch of superior mesenteric artery apart from the obliteration of inferior mesenteric artery leads to the formation of the second pouch. The interposed segment of normal colon remains unaffected as its vascular supply through the middle colic branch of the superior mesenteric artery remains patent [4]. The proximal end of interposed colon in the present case probably became atretic in late gestation due to some vascular accident (probably occlusion of a branch of middle colic artery). This is the 4th case of type 5 CPC reported in world literature [4, 5].

## Footnotes

**Source of Support:** Nil

**Conflict of Interest:** None

